# Impact of tumor contact surface area on collecting system entry in robot-assisted partial nephrectomy: a retrospective analysis

**DOI:** 10.1186/s12894-023-01247-0

**Published:** 2023-05-08

**Authors:** Tatsuya Umemoto, Masanori Hasegawa, Soichiro Yuzuriha, Tatsuo Kano, Takahiro Ogawa, Masayoshi Kawakami, Mayura Nakano, Hakushi Kim, Masahiro Nitta, Yoshiaki Kawamura, Sunao Shoji, Ryuichi Mizuno, Akira Miyajima

**Affiliations:** 1 Department of Urology, Tokai University School of Medicine, 143 Shimokasuya, Isehara, 259-1193 Kanagawa Japan; 2 Department of Urology, Keio University School of Medicine, Tokyo, Japan

**Keywords:** R.E.N.A.L nephrometry score, Renal cell carcinoma, Robot-assisted partial nephrectomy, Ureteral catheter

## Abstract

**Background:**

Collecting system entry in robot-assisted partial nephrectomy may occur even in cases showing a low N factor in the R.E.N.A.L nephrometry score. Therefore, in this study, we focused on the tumor contact surface area with the adjacent renal parenchyma and attempted to construct a novel predictive model for collecting system entry.

**Methods:**

Among 190 patients who underwent robot-assisted partial nephrectomy at our institution from 2015 to 2021, 94 patients with a low N factor (1–2) were analyzed. Contact surface was measured with three-dimensional imaging software and defined as the C factor, classified as C1, < 10 cm [2]; C2, ≥ 10 and < 15 cm [2]; and C3: ≥ 15 cm [2]. Additionally, a modified R factor (mR) was classified as mR1, < 20 mm; mR2, ≥ 20 and < 40 mm; and mR3, ≥ 40 mm. We discussed the factors influencing collecting system entry, including the C factor, and created a novel collecting system entry predictive model.

**Results:**

Collecting system entry was observed in 32 patients with a low N factor (34%). The C factor was the only independent predictive factor for collecting system entry in multivariate regression analysis (odds ratio: 4.195, 95% CI: 2.160–8.146, p < 0.0001). Models including the C factor showed better discriminative power than the models without the C factor.

**Conclusions:**

The new predictive model, including the C factor in N1-2 cases, may be beneficial, considering its indication for preoperative ureteral catheter placement in patients undergoing robot-assisted partial nephrectomy.

**Supplementary Information:**

The online version contains supplementary material available at 10.1186/s12894-023-01247-0.

## Background

Partial nephrectomy (PN) has been the standard treatment for clinical stage T1a renal cell carcinoma (RCC). [1–3] Robot-assisted partial nephrectomy (RAPN) has recently become the standard operative method for these cases, and the indications for RAPN have been expanded to include even some T1b RCCs. [4, 5] The R.E.N.A.L nephrometry score (RNS) is a scoring system developed by Kutikov and Uzzo for standardized assessment of the anatomical features of renal tumors. [6] The RNS has been reported to be related to PN perioperative outcomes, complications, and ischemic time. [7–9] This scoring system is based on five factors characterizing the anatomy of a renal tumor. Among these, the R score represents the maximal diameter of the tumor and is scored as follows: 1, ≤ 4 cm; 2, > 4 to < 7 cm; and 3: ≥ 7 cm. The E score represents the properties of the tumor (exophytic/endophytic) and is scored as follows: 1, ≥ 50% endophytic; 2, > 50% endophytic; and 3, entirely endophytic. The N score represents the tumor’s proximity to the renal sinus or collecting system and is scored as follows: 1, ≥ 7 mm; 2, > 4 to < 7 mm; and 3, ≤ 4 mm. The A factor represents the tumor position (anterior or posterior) but is not scored. The L factor indicates the tumor location relative to the polar line and is scored as follows: 1, entirely above or below the polar line; 2, crossing the polar lines; and 3, > 50% of the mass crossing the polar line or located entirely between the polar lines. The N factor has been reported to be useful for evaluating the risk for collecting system entry (CSE), one of the potential complications of PN. [10] Therefore, ureteral catheter placement is considered for the prevention of postoperative urine leakage in cases with a high risk of CSE, such as those with an N score of 3. Nevertheless, we have experienced CSE in cases with low N factor scores of 1 or 2, and there was no clear information available regarding the risk factors for CSE.

With the recent advancements in imaging techniques, volume measurements can be easily obtained from two-dimensional images, and three-dimensional (3D) reconstruction has been commonly utilized in clinical practice. In this regard, the psoas muscle volume was shown to influence the development of inguinal hernia after robot-assisted radical prostatectomy and predict the prognosis of patients with upper urinary tract urothelial carcinoma who underwent radical nephroureterectomy. [11, 12] The contact surface (CS) area is the contact area between the renal tumor and the renal parenchyma, which can be easily calculated using 3D analysis software. Previous studies have reported that a higher CS is related to a longer warm ischemic time, higher estimated blood loss, longer duration of hospitalization, and decreased renal function. [13] However, to our knowledge, they did not reveal the relationship between the CS and CSE. For tumors showing a large contact area with the renal parenchyma, surgeons set the cutting line of the tumor wider because of the difficulty in imaging the tumor depth and concerns regarding a positive surgical margin. Therefore, we hypothesized that a large CS would increase the risk of CSE and considered the possibility of using CS measurements as a risk factor for CSE. To this end, we focused on tumor size (RNS-R factor), endophytic property (RNS-E factor), location (RNS-L factor), and the CS and aimed to construct a novel predictive model for CSE during RAPN.

## Methods

### Patients

We retrospectively analyzed 190 patients who underwent RAPN from May 2015 to July 2021 at our hospital and extracted and included the data for 94 patients with N factor scores of 1–2. The patient characteristics are shown in Supplementary Table 1. The average age and body mass index (BMI) were 63.6 years and 24.2 kg/m^2^, respectively. The study included more men than women (74 vs. 20) and more patients with left-sided than right-sided (51 vs. 43) tumors. The tumor size was 21.2 ± 8.3 mm (T1a: 91 cases, T1b: 3 cases).

### R.E.N.A.L nephrometry score

The average RNS total score in this study was 5.8 ± 1.3. The R score was almost 1 in 90/94 cases. Since RAPN is mainly intended for pT1a tumors smaller than 4 cm, size cannot be accurately evaluated with the original R score in RNS. Therefore, we defined a new R scoring factor named “modified R (mR)” in this study and scored it as follows: 1, < 20 mm; 2, ≥ 20 and < 40 mm; and 3, ≥ 40 mm.

### Surgical procedures

RAPN was performed for T1a-T1b RCCs by seven surgeons using the da Vinci surgical system (da Vinci XI, Intuitive Surgical Inc, California, USA). All operators had sufficient experience with robot-assisted radical prostatectomy and laparoscopic partial nephrectomy. Each surgeon had experience of 106, 50, 22, 20, 16, 8, and 5 cases of RAPN. The surgical approach (transperitoneal or retroperitoneal) was determined at the conference based on the tumor height and location (anterior or posterior). Ureteral catheter placement was performed in all cases with a high N factor score of 3. Conversely, the indications for ureteral catheter placement were discussed at the preoperative conference in cases with an N factor score of 1 − 2. Ureteral catheter placement was performed in all cases except in those where CSE was predicted to not occur based on multiple surgeons’ opinions. Under general anesthesia, a 6-Fr single-J ureteral stent was inserted into the renal pelvis using a cystoscope. Tumor enucleation was only performed in some N3 cases, in which there was contact with the renal hilum. The tumor was resected with a 2-mm surgical margin. Therefore, the procedure of enucleation was not included in this study. After tumor removal, in cases involving the placement of a ureteral catheter, a retrograde injection of indigotindisulfonate sodium through the ureteral catheter was performed to identify the position of CSE. If CSE was detected, it was sutured with 3 − 0 V-Lok sutures. After central suturing, indigotindisulfonate sodium was injected again to confirm complete closure. The renal artery was de-clamped after applying hemostasis sutures, and parenchymal sutures were applied with a 3 − 0 monofilament absorbent thread. Drain tubes were placed in all cases to remove surgery fluid and diagnose postoperative complications, such as bleeding or urine leakage. The ureteral catheter was removed on postoperative days 3–4 when CSE was present and immediately after surgery in cases without CSE.

### Estimation of the contact surface by using 3D images

**Fig. 1:**
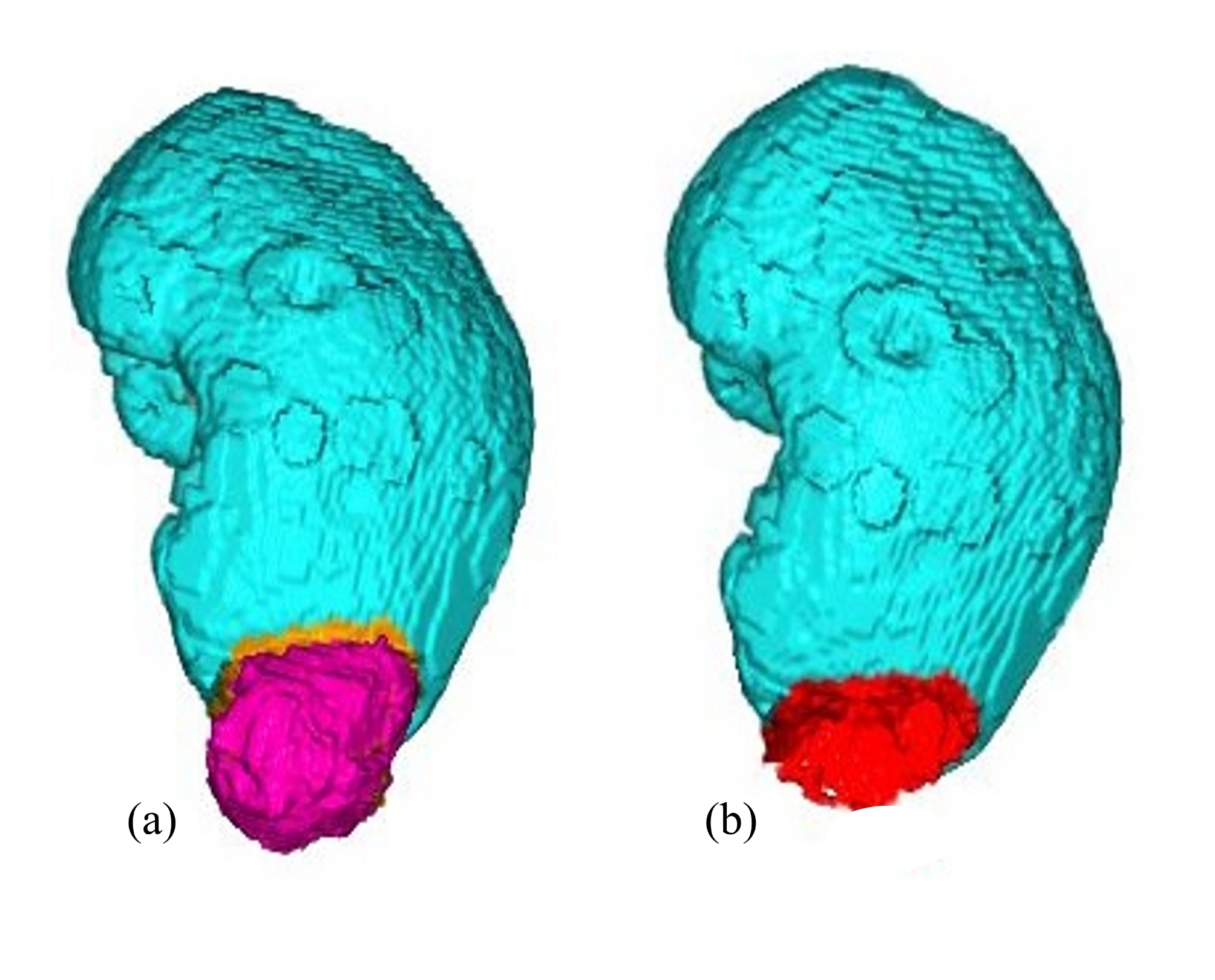
Three-dimensional tumor quantification based on CT images. The figure shows a renal cell carcinoma (RCC) of the left kidney using coronal CT. The resection margin was stained yellow, and the RCC was stained purple (**a**). Image of the contact surface after RCC resection (red, **b**)

CT scans for evaluation of the RCC were performed preoperatively in all patients. The CS was quantified from CT data by using the 3D image analysis system, Synapse Vincent ver.4® (Fujifilm, Tokyo, Japan). This system automatically determined the contraction of the kidney and tumor and measured the tumor volume. In the next step, the software simulated the removal of the tumor from the renal parenchyma and determined the post-removal area of the renal parenchyma as the CS. In this study, CS was measured with a resection margin of 2 mm on imaging. The working steps and 3D images are shown in Fig. 1a-b. Figure 1a shows the RCC and the 2-mm surgical margin stained in yellow. Figure 1b shows the CS after RCC resection in red.

### Statistical analysis

The C factor was scored as follows: C1, < 10 cm [2]; C2, ≥ 10 and < 15 cm [2]; and C3, ≥ 15 cm [2]. Univariable and multivariable logistic regression analyses were performed to identify which combination of factors among RNS-mR, E, L, and C could predict a positive CSE. The CSE-predicting abilities of the models with and without the C-factor were compared (mRE vs. mREC, mREL vs. mRELC). The chi-square test and Mann–Whitney U test were used as categorical and continuous variables to evaluate group differences. Significance was defined as p < 0.05. All statistical analyses were performed using SPSS software, version 26® (IBM, Japan) and EZR (Saitama Medical Center, Jichi Medical University, Saitama, Japan), a modified version of R commander (R Foundation for Statistical Computing, Vienna, Austria) designed to add statistical functions frequently used in biostatistics.

### Ethics approval

This study was performed in line with the principles of the Declaration of Helsinki. Approval was granted by the Ethics Committee of Tokai University (approval number; 21R101).

## Results

### Perioperative results

The perioperative results are presented in Table 1. The tumor volume and CS were 7.2 ± 9.6 mL and 11.8 **±** 4.9 cm [2], respectively, in accordance with the measurements obtained by Synapse Vincent®. The operative time, surgeon console time, and warm ischemic time were 176.3 ± 45.3 min, 110.7 ± 40.5 min, and 751 (310–2571) s, respectively. The estimated blood loss was 41 mL (2–400 mL). Preoperative ureteral catheter placement was performed in 60 cases (63.8%), and CSE was not observed in 30 of these cases (30/60, 50%). On the other hand, CSE was observed in 32 cases (32/94, 34%); thus, 2 cases showed positive findings for CSE without catheter placement. There was no postoperative urine leakage in this cohort.

**Table 1:** Perioperative findings

Factors	Total (n = 94)
Operative time (min), mean ± SD	176.3 ± 45.3
Console time (min), mean ± SD	110.7 ± 40.5
Warm ischemic time (s), median (range)	751 (310–2571)
Estimated blood loss (mL), median (range)	41 (2–400)
Tumor volume (mL), mean ± SD	7.2 ± 9.6
Tumor contact surface area (cm [2]), mean ± SD	11.8 ± 4.9
R.E.N.A.L nephrometry score, mean ± SD	5.8 ± 1.3
mRE score, mean ± SD	2.6 ± 0.6
mREC score, mean ± SD	4.3 ± 1.1
Ureteral catheter placement, n (%)	60 (63.8%)
Collecting system entry, n (%)	32 (34%)
Postoperative urine leakage, n (%)	0 (0%)

### Analysis of the CSE and non-CSE groups

The patients were categorized into CSE and non-CSE groups, and their preoperative and perioperative factors, such as age, sex, laterality, BMI, tumor size, tumor volume, CS, RNS score, operative time, console time, and warm ischemic time, were analyzed. The groups showed no significant differences in age, sex, laterality, BMI, operating time, and console time. The CSE group involved a significantly longer warm ischemic time, larger tumor size, larger tumor volume, larger CS, and higher RNS score (Table 2).

**Table 2:** Comparison of preoperative and perioperative results for collecting system entry

	CSE (n = 32)		Non-CSE (n = 62)	P-value
Age	62		64.5	0.208
Sex				
Male Female	8 24		50 12	0.526
Side				
Right Left	15 17		28 34	0.874
Body mass index	24.7		23.9	0.158
Contact surface (cm [2])	14.7		10.2	< 0.001
Tumor volume (mL) Tumor size (mm) RNS score	12.6 25.8 6.3		4.3 18.8 5.6	< 0.001 < 0.001 0.019
Operative time (min)	179.5		174.6	0.193
Console time (min)	110.8		110.6	0.439
Warm ischemic time (s)	1092.9		726.8	< 0.001

### Analysis of factors affecting CSE

**Fig. 2:**
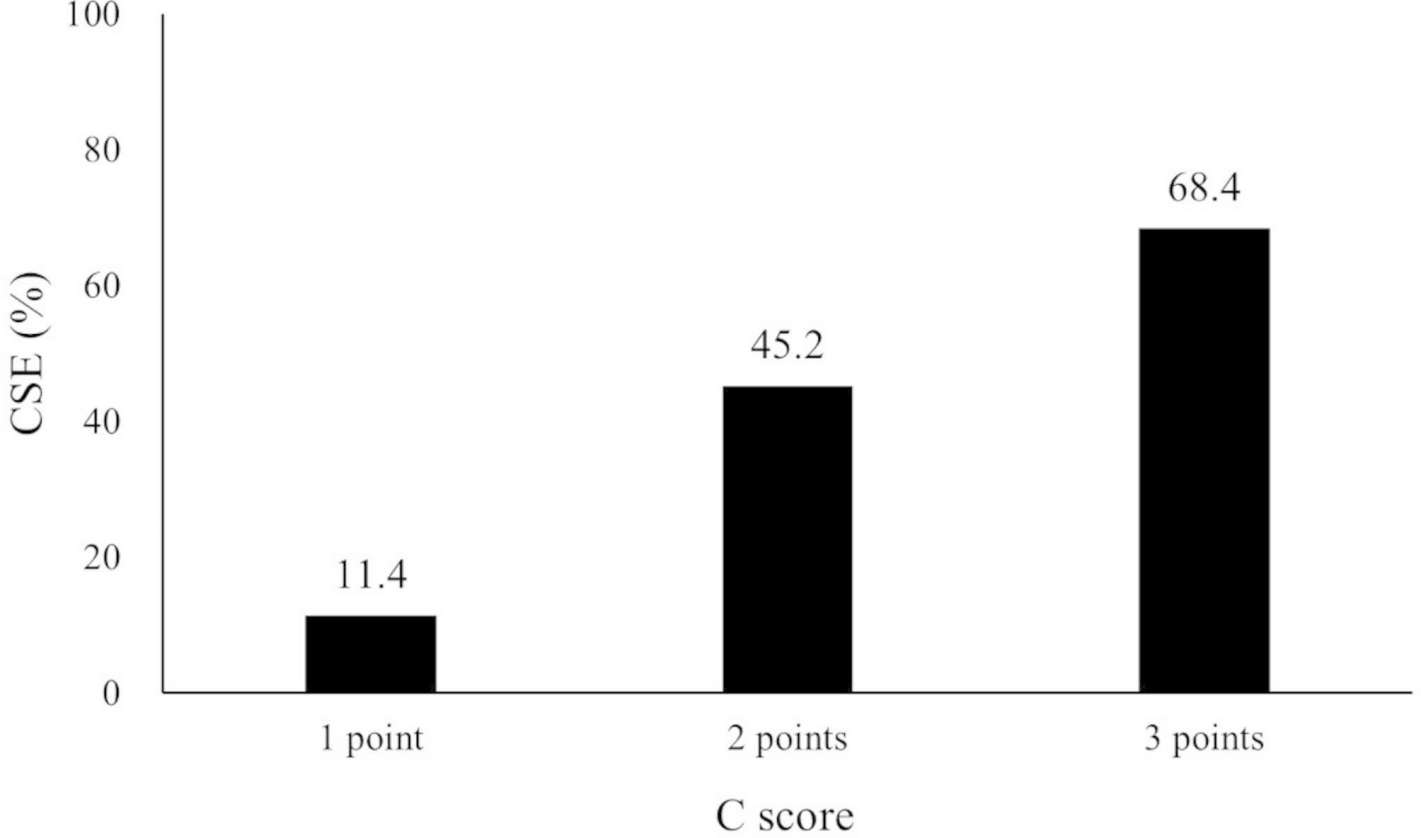
Probability of collecting system entry (CSE) based on the C-score

We examined the relationship between the C score and CSE (Fig. 2). In cases with a C score of 1, CSE showed a low incidence of 11.4%. However, C scores of 2 and 3 corresponded to CSE incidence rates of approximately 50% and 80%, respectively. Univariate and multivariable analyses were performed using RNS with mR, E, L, and C as the factors. In the univariate analysis, high mR and C scores showed significant interactions with the occurrence of CSE (p = 0.004 and p < 0.0001, respectively, Table 3). In multivariable regression, only the C factor was significantly associated with the occurrence of CSE (p < 0.0001).

**Table 3:** Univariate and multivariate analysis of the factors related to CSE

	Univariate analysis			Multivariate analysis		
	OR	95% CI	p-value	OR	95% CI	p-value
R factor (continuous)	3.221	1.450–7.155	0.004	1.682	0.659–4.289	0.277
E factor (continuous)	1.083	0.542–2.166	0.821	0.855	0.347–2.104	0.732
L factor (continuous)	1.267	0.764-2.100	0.359	1.382	0.762–2.505	0.287
C factor (continuous)	4.195	2.160–8.146	< 0.001	3.824	1.808–8.090	< 0.001

### Construction of novel predictive models for CSE

**Fig. 3:**
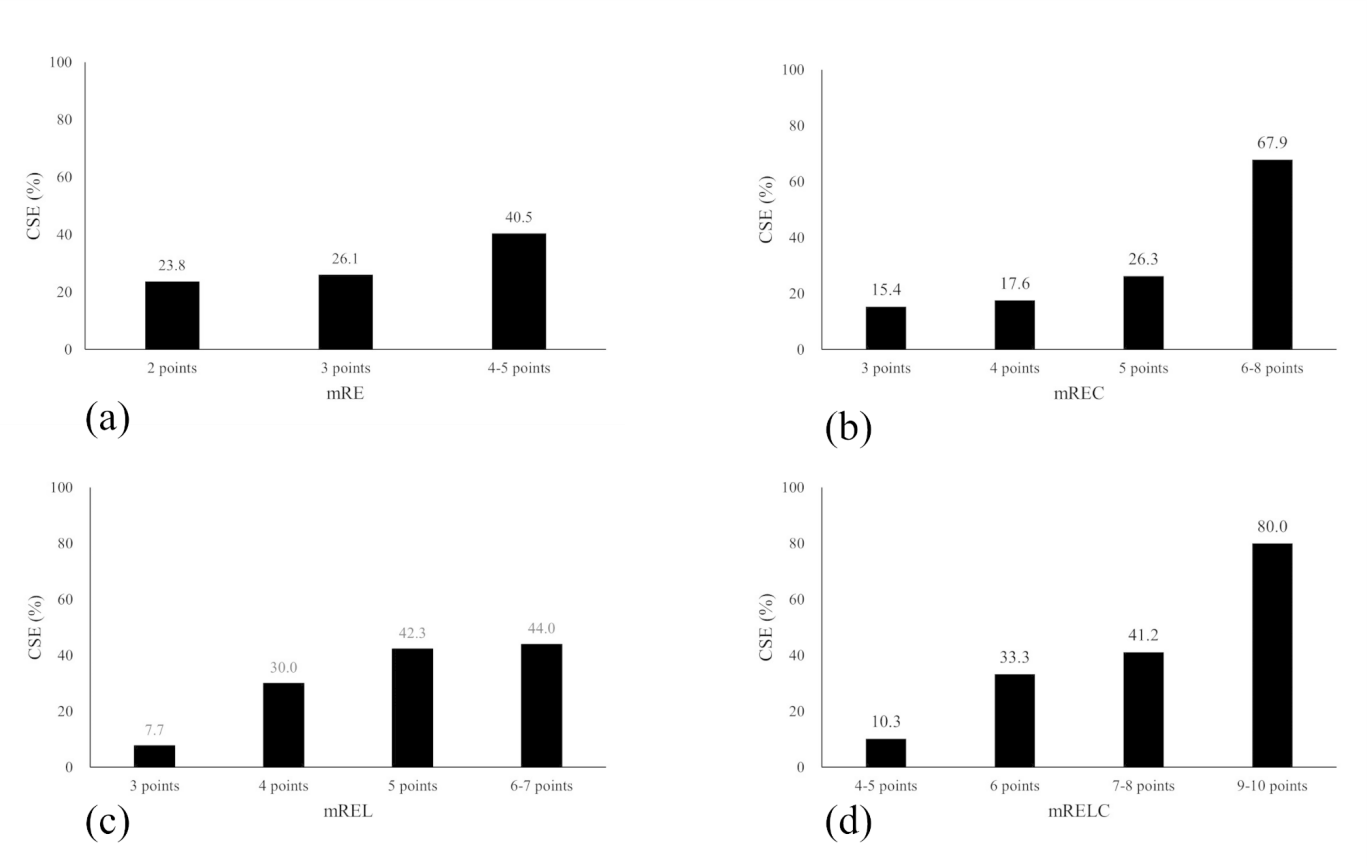
Probability of collecting system entry (CSE) based on the mRE score (**a**), mREC score (**b**), mREL score (**c**), and mRELC score (**d**)

We constructed and compared modified nephrometry scoring systems with or without the C factor, i.e., mR + E + C (mREC) versus mR + E (mRE), and mR + E + L + C (mRELC) versus mR + E + L (mREL) (Fig. 3a-d). The probability of CSE was 15% for an mREC score of 3 points, and it increased to 67.9% for an mREC score of 6–8 points. In contrast, with the mRE, the probability of CSE was only 40% at the maximum mRE score of 4–5 points. Similarly, the probability of CSE was 10% for an mRELC score of 4–5 points, and it increased to 80% for an mRELC score of 9–10 points, while the probability of CSE was only 40% for the highest mREL score of 6–7 points.

**Fig. 4:**
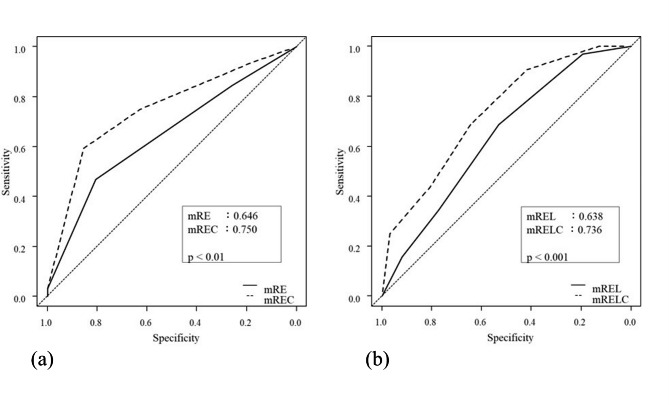
The figure shows a comparison of the scores for predicting collecting system entry (CSE) using the C-index. mREC versus mRE (**a**) and mRELC versus mREL (**b**)

We additionally analyzed the accuracy of models for predicting CSE by using C-index values. The models’ C-index values were 0.750 (mREC), 0.736 (mRELC), 0.646 (mRE), and 0.636 (mREL) (Fig. 4a-b). The mREC and mRELC models, which included the C factor, were significantly better than the mRE (p < 0.01) and mREL (p < 0.01) models, respectively.

## Discussion

The present study retrospectively investigated the influence of CS on the occurrence of CSE after RAPN in cases with a low RNS N score (N1-2). CS was measured using 3D imaging software and enhanced CT scan images. The average CS was 11.8 ± 4.9 cm [2]. The C factor was the only significant factor influencing CSE in the multivariable regression. We then attempted to develop novel models using the C factor for predicting CSE. The mREC and mRELC models, which included the C factor, were significantly better than the corresponding models without the C factor (i.e., the mRE and mREL models, respectively) for predicting CSE. The C-index values were 0.750 (mREC), 0.736 (mRELC), 0.646 (mRE), and 0.636 (mREL), which suggested that the addition of the C score to the RNS improved the accuracy of predicting CSE. Since 50% of patients in the preoperative ureteral catheter placement group did not show CSE, the medical costs and degree of treatment invasiveness could be reduced if CSE could be predicted preoperatively.

The RNS is used to predict the difficulty of partial nephrectomy, and higher scores have been reported to correlate with the ischemic time, postoperative renal function, operative time, and duration of hospitalization. [14, 15] The N factor of the RNS represents the tumor’s proximity to the collecting system or sinus, and high scores for the N factor have been correlated with the occurrence of CSE. [10] An N factor score of 3 represents a distance less than 4 mm, which indicates a high probability of CSE. Consistent with this finding, the incidence of positive CSE in cases with an N score of 3 in our cohort was 71% (70/98 cases). However, CSE can often occur during RAPN in cases with low N scores of 1–2. Although the higher incidence of CSE in cases with high N scores is intuitively easy to understand, no previous studies, to our knowledge, have analyzed the reasons influencing the occurrence of CSE in cases with a low N score. Therefore, we aimed to identify a novel factor for predicting positive CSE. The CS represents the contact area between the renal tumor and renal parenchyma, and it can be easily calculated using 3D analysis software by generalizing 3D software in clinical practice. In previous reports, a higher CS was associated with a longer warm ischemic time, higher estimated blood loss, longer duration of hospitalization, and decreased renal function. [13] However, considering the lack of studies evaluating the influence of CS on the occurrence of CSE, we analyzed this influence by evaluating the relationship between CSE and CS in the present study.

This analysis revealed that the most important factor influencing the occurrence of CSE in patients with a low N score is not a large tumor diameter or the endophytic nature of the tumor but a large CS. One of the reasons for this finding could be that the resection line “curve” becomes longer when a tumor shows a large contact area, which contributes to a larger CS. Additionally, a large CS increases the difficulty of imaging the cutting line, necessitating larger and deeper resection to avoid a positive surgical margin.

Urine leakage is one of the complications of PN that are related to incomplete repair of the CSE. [16–19] The incidence of urine leakage has been reported to be approximately 1–5% in some cohorts, [17, 20, 21] and the available options for urine leakage include observation, ureteral drainage, percutaneous drainage, and surgical interventions. [22–24] Preoperative ureteral catheterization has been widely used to recognize and prevent the occurrence of urine leakage. [25] However, the use of ureteral catheter placement in all PN cases is questionable because of the increased cost and extended operation time. [26].

At our hospital, preoperative ureteral catheter placement was performed in all N3 cases, but in N1-2 cases, it was only performed when deemed necessary at the preoperative conference. However, there were no clear criteria for ureteral catheter placement, and approximately 50% of the cases involving ureteral catheter placement in our cohort did not have CSE; thus, a clearer catheter placement standard was thought to be necessary considering the medical costs and complications associated with catheter placement (including ureteral perforation and hematuria). Thus, the new scoring system to predict CSE using the C factor may be useful for determining cases requiring ureteral catheter placement.

We acknowledge that the present study has several limitations. The study population was small and examined retrospectively. Considering each surgeon’s experience, it is assumed that there was variation in technical capabilities, and the current study may not be applicable to other population due to the difference of overall experience of the team. [27] In addition, CS measurements required 3D image software, like Synapse Vincent®. Thus, our results need to be validated using larger cohorts in future studies.

It has been recognized that a discussion of whether a ureteral catheter insertion is required or not when CSE occurs is essential. In order to perform RAPN safely, it is important to be attentive when finding the entry point on the cutting surface and confirming a satisfactory closure after suturing. This model may thus help us to recognize CSE preoperatively in N1-2 cases, and we believe this may help surgeons, especially novice surgeons, perform RAPN safely.

## Conclusions

In conclusion, we investigated the effect of the CS area in predicting CSE in a patient with a low N score (N1-2) on the RNS who underwent RAPN. The findings showed that the C score was most strongly associated with CSE among all factors of the RNS. The mREC and mRELC scores, which included the C factor, showed high accuracy in predicting CSE, and preoperative ureteral catheter placement may be considered for cases with high mREC and mRECL scores.

## Electronic supplementary material

Below is the link to the electronic supplementary material.

Additional File 1: Patient characteristics

## Data Availability

The datasets generated and/or analyzed during the current study are not publicly available because the data also form part of an ongoing study but are available from the corresponding author upon reasonable request.

## List of Abbreviations

CS: The Contact Surface
CSE: Collecting system entry
PN: Partial nephrectomy
RCC: Renal cell carcinoma
RNS: The R.E.N.A.L nephrometry score
