# Supplementary material for: Impact of tumor contact surface area on collecting system entry in robot-assisted partial nephrectomy: a retrospective analysis
Source: BMC Urol. 2023 May 8;23:85. doi: 10.1186/s12894-023-01247-0 (PMC10165753; doi:10.1186/s12894-023-01247-0)
Supplement: Supplementary file 1 — Additional File 1: Patient characteristics [file 12894_2023_1247_MOESM1_ESM.docx]

**Supplemental Table 1.** Patient characteristics

| Factors | Total (n=94) |
| --- | --- |
| Sex  Male  Female | 74 (78.7 %)  20 (21.3 %) |
| Side  Right  Left | 43 (45.7 %)  51 (54.3 %) |
| Age (years), mean **±** SD | 63.6 **±** 11.8 |
| Body mass index, mean **±** SD | 24.2 **±** 4.2 |
| Tumor size (mm), mean **±** SD | 21.2 ± 8.3 |
